# Abundance of ultramicro inversions within local alignments between human and chimpanzee genomes

**DOI:** 10.1186/1471-2148-11-308

**Published:** 2011-10-19

**Authors:** Yuichiro Hara, Tadashi Imanishi

**Affiliations:** 1 Biomedicinal Information Research Center (BIRC), National Institute of Advanced Industrial Science and Technology (AIST), Aomi 2-4-7, Koto-ku, Tokyo, Japan

## Abstract

**Background:**

Chromosomal inversion is one of the most important mechanisms of evolution. Recent studies of comparative genomics have revealed that chromosomal inversions are abundant in the human genome. While such previously characterized inversions are large enough to be identified as a single alignment or a string of local alignments, the impact of ultramicro inversions, which are such short that the local alignments completely cover them, on evolution is still uncertain.

**Results:**

In this study, we developed a method for identifying ultramicro inversions by scanning of local alignments. This technique achieved a high sensitivity and a very low rate of false positives. We identified 2,377 ultramicro inversions ranging from five to 125 bp within the orthologous alignments between the human and chimpanzee genomes. The false positive rate was estimated to be around 4%. Based on phylogenetic profiles using the primate outgroups, 479 ultramicro inversions were inferred to have specifically inverted in the human lineage. Ultramicro inversions exclusively involving adenine and thymine were the most frequent; 461 inversions (19.4%) of the total. Furthermore, the density of ultramicro inversions in chromosome Y and the neighborhoods of transposable elements was higher than average. Sixty-five ultramicro inversions were identified within the exons of human protein-coding genes.

**Conclusions:**

We defined ultramicro inversions as the inverted regions equal to or smaller than 125 bp buried within local alignments. Our observations suggest that ultramicro inversions are abundant among the human and chimpanzee genomes, and that location of the inversions correlated with the genome structural instability. Some of the ultramicro inversions may contribute to gene evolution. Our inversion-identification method is also applicable in the fine-tuning of genome alignments by distinguishing ultramicro inversions from nucleotide substitutions and indels.

## Background

Chromosomal inversion, a type of genetic rearrangement involving the inversion of a chromosome segment, is one of the most important causes of genomic changes. Inversions have been identified as phylogenetic signatures since the first third of the twentieth century [1] and are thought to have affected phenotypic evolution [2]. While large-size inversions (macroscopic inversions), microscopically-detectable and/or visible in genetic maps, were identified early on [1,3], the recent abundance of genomic sequences and progress in sequence analysis has enabled the extensive detection of inversions of various sizes in genomes. In particular, comparative genomics between populations and between closely related species have revealed the occurrence of numerous inversions in genomes including small-size (microscopic) inversions [4-6]. More than 1,500 inversions varying in length from 23 bp to 62 Mb occur in the human and chimpanzee genomes, suggesting that inversions are common mechanisms for differentiating genomes [6]. Although several methods have been developed to identify these inversions, they focus only on the macroscopic or microscopic inversions which are inversions large enough to be detected as a single alignment or a string of local alignments [6].

Some inversions may be too small to be identified even as a local alignment block. Such ultramicro inversions are extremely difficult to detect using existing methods because they may be hidden within the local alignments of BLAST or other popular alignment softwares. These inversions are very short such that the alignment extends beyond them allowing mismatches and gaps. In such cases, the ultramicro-inverted regions are treated as small arrays of mismatches and gaps within the local alignments. The degree of overlooking the ultramicro inversions hidden within the local alignments would be higher within the alignment between closely related genomes, because mismatches in short regions are negligibly small for highly similar alignments longer than ten kilobases.

These ultramicro inversions would be aligned with mismatches and gaps more frequently than would a random distribution of such differences. Generally, mismatches and gaps within alignments account for nucleotide substitutions and insertions and deletions (indels), respectively. However, mismatches and gaps generated at inverted sites are a result of erroneous alignments. Whether or not differences in the alignments are caused by nucleotide substitutions and indels is apparently unclear. Thus, it is difficult to obtain information about ultramicro inversions from the local alignments themselves. Identifying ultramicro inversions within the alignments would be necessary for distinguishing the mismatches and gaps caused by nucleotide substitutions and indels from those caused by inversions.

The human genome is different from the chimpanzee genome, at 1.2% of sequence mismatches [7] and 5% of sequence mismatches plus gaps [8]. Some of these differences are assumed to play important roles in the phenotypic evolution of the human lineage. Furthermore, macroscopic inversions are one of the major mechanisms of differentiating species [2]. For example, pericentric inversion is one type of large genomic rearrangements which distinguishes the human karyotype from that of the chimpanzee [9,10], implying that such inversions are one of the important causes of speciation. Ultramicro inversions may also be spread across the human and chimpanzee genomes because the size distribution of the macroscopic and microscopic inversions decays as the size of the inversions increases [6]. In addition, the differences in the human-chimpanzee alignments caused by inversions raise the average differences between the human and chimpanzee genomes. In order to examine the impact of ultramicro inversions on the genome alignment, we developed a method for identifying ultramicro inversions within the alignments between the human and chimpanzee genomes. We first generated 2.41 Gb of one-to-one (i.e., orthologous) alignments between the human and chimpanzee genomes using the G-compass pipeline [11,12], and identified inversions in each local alignment. Subsequently, we examined the relationships of ultramicro inversions with the structural features of the human genome to determine the molecular mechanisms of the inversions. Furthermore, we examined biologically functional segments to infer the effects of the inversions on the phenotypic evolution of the human lineage.

## Results

### Simulation

Firstly, we defined ultramicro inversions as the inverted regions buried within local alignments. With this definition, most of the "ultramicro" inversions are expected to be smaller than the "microscopic" inversions which are identified as a single alignment or a string of local alignments. Within the local alignments, the ultramicro inversions would be misaligned forwardly. We developed a method for identifying such ultramicro inversions hidden within regions of local pairwise-alignments rich in mismatches and gaps. In such regions, erroneously aligned ultramicro inversions would possess high density of mismatches and gaps. Assuming that the sequence differences are spread across the genome following a negative binominal distribution, we determined if these regions could be aligned inversely with greater similarity than the forward alignments (See Methods). A simulation was conducted in order to test the strength and accuracy of this algorithm using the Indelible program [13] for evolving random sequences. Eleven sets of pairwise nucleotide alignments were generated allowing the creation of indels, each consisting of 100,000 pairs of 5,000 bp random nucleotide sequences, with parameters (e.g., differences and base composition) equivalent to the human-chimpanzee genome alignments. A short (5-50 bp) segment with fixed length was randomly chosen and inverted in one sequence of every pair. Re-aligned pairwise sequences were then subjected to the inversion identification.

**Figure 1:**
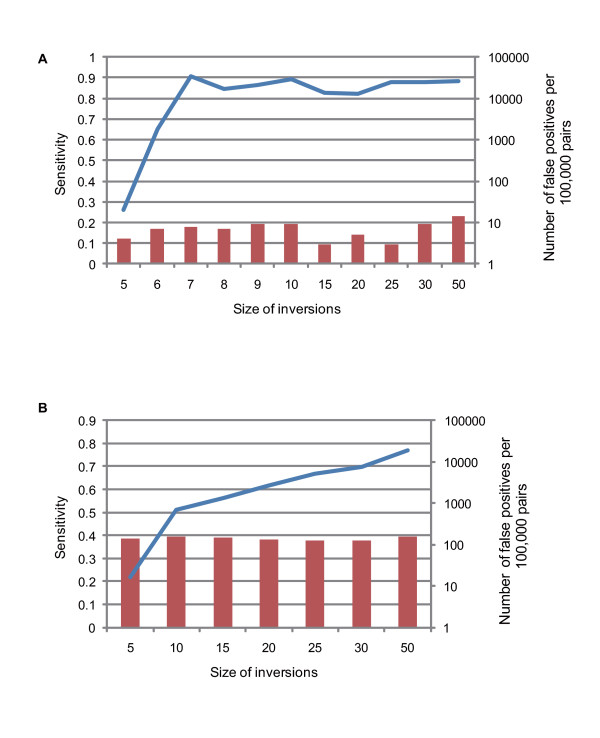
**Results of the ultramicro inversion identification**. Sensitivities (blue lines) and numbers of false positives (red bars) for the simulations, assuming sequence parameters equivalent to the alignments between the human and chimpanzee genomes (A) and those of the AT-exclusive condition (B).

Through the identification of inversions, the sensitivity of the algorithm was found to approximately range from 0.82 to 0.91, except for the 5 and 6 bp inversions; 0.261 for 5 bp and 0.651 for 6 bp inversions (Figure 1A). Hence, this method is particularly useful for identifying all inversions except the extremely short ones. In addition, only 14 false positives were found in a set of 100,000 pairs of sequences (Figure 1A), suggesting a very low false-positive rate. Only 67 false positives were expected in the human-chimpanzee alignments consisting of 2.4 Gb of alignment sites.

### Identification of inversions between the human and chimpanzee genomes

**Figure 2:**
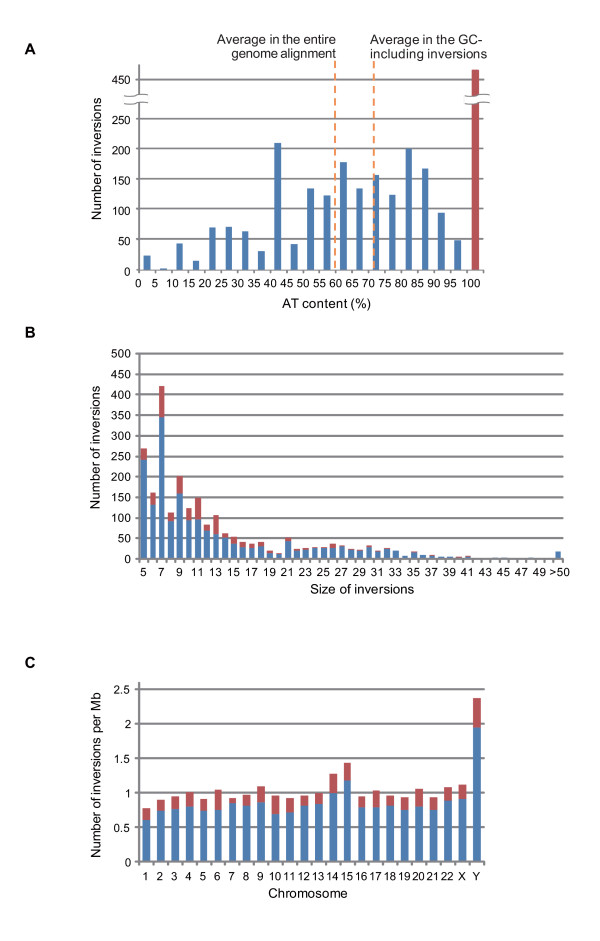
**AT content, size, and chromosomal distribution of ultramicro inversions**. Distributions of ultramicro inversions over the ranges of AT content (A), sizes in nucleotides (B), and chromosomes (C). The red and blue bars represent the numbers of AT-exclusive and GC-including inversions. In (A), the average of AT content in the entire genome alignment between the human and chimpanzee genomes and that in GC-including inversions are also shown.

We detected 2,377 ultramicro inversions hidden within the one-to-one alignments between the human and chimpanzee genomes. Interestingly, 461 inversion segments consisted of adenine and thymine exclusively (AT-exclusive inversions) (Table 1 and Figure 2A; extensive information on ultramicro inversions in the TSV format is available at http://hinv.jp/g-compass/2011hara/index.html). In addition, AT content of the inversions excluding the AT-exclusive segments was higher (72.0%) than the average AT content of the entire human-chimpanzee alignments (59.3%) (Figure 2A), suggesting that ultramicro inversions preferentially occurred in AT-rich regions. The AT-exclusive regions possess considerably different conditions from the other regions to the extent of AT content and thus would show different power for the inversion identification from that assumed in the simulation in previous subsection. In order to validate the strength and accuracy of the inversion identification methods for the AT-exclusive regions, we conducted a simulation under the prior conditions except for different base compositions of the AT-exclusive regions (50% adenine and 50% thymine). Sensitivity for searching for the true ultramicro inversions in simulation in the AT-exclusive condition was less than that in the initial condition and, as well as the initial condition, increased with increasing inversion size from 0.218 for 5 bp inversion to 0.768 for 50 bp inversion (Figure 1B). False positives were greater in the AT-exclusive conditions than in the initial simulation condition with less than 160 false positives in the 500 Mb AT-exclusive simulation set (Figure 1B). Although 760 or less false positives were expected in the 2.4 Gb AT-exclusive alignments, which was as large as the human-chimpanzee genome alignments, the number of false positives in the human-chimpanzee genome alignments may have been much lower than 760 since the AT blocks constitute small fractions of the genome. Blocks consisting of a series of at least 5 bp of adenines and thymines were summed at approximately 120 Mb in the human genome, in which all the 461 AT-exclusive inversions were included, indicating that less than 38 false positives of the AT-exclusive inversions could be expected in the human-chimpanzee alignments.

**Table 1:** Overview of ultramicro inversions within alignments between the human and chimpanzee genomes.

Inversion classification and the lineage of inversion events	GC-including	AT-exclusive	Total
Ultramicro inversions	1926	461	2377
Inversions obtained with the support of phylogenetic profiles	1218	206	1424
Human lineage	435	44	479
Chimpanzee lineage	758	153	911
Human-Gorilla lineage*	14	6	20
Chimpanzee-Gorilla lineage*	11	3	14

*Subject to incomplete lineage sorting among human, chimpanzee, and gorilla.

The size of ultramicro inversions between the human and chimpanzee genomes ranged from 5 to 125 bp (Figure 2B and the extensive information file), and the distribution of their lengths, which was classified into three characteristics, showed a peculiar shape. While this distribution basically decayed in a fashion similar to the macroscopic and microscopic inversions between the human and chimpanzee genomes [6], extremely short inversions were less frequent (Figure 2B). This is because our identification method was less sensitive to extremely short inversions as shown in the simulation results. The most peculiar feature was that among the ≤13 bp inversions, ultramicro inversions with odd number lengths were more frequent than those with even number lengths. The frequency of ultramicro inversions seemed to be independent of whether the inversion length was odd or even in the simulations (Figure 1A). This implies that different shapes of the distributions of the odd and even numbered-inversion lengths were not likely because of the internal cause of the identification algorithm. In addition, we found a small peak around 20 bp in the size distribution of the ultramicro inversions.

While the ultramicro inversions as well as the macroscopic and microscopic inversions were spread throughout the human genome [6], the density of inversions was significantly different on chromosome Y compared with that on the autosomes (Figure 2C). Autosomes averaged 0.196 ± 0.0519 AT-exclusive and 0.807 ± 0.112 guanine and cytosine-including (GC-including) inversions per Mb. However, chromosome Y possessed more frequent inversions: 0.422 AT-exclusive inversions (p < 1.00 × 10^-5^) and 1.94 GC-including inversions (p < 1.00 × 10^-5^) per Mb. In contrast, the numbers of AT-exclusive and GC-including inversions on chromosome X (0.215 and 0.906 per Mb, respectively) were not significantly different from those on the autosomes (p = 0.335 and 0.380, respectively). In addition, the proportions of the inversion ratios between chromosome Y and autosomes (2.16 times for AT-exclusive and 2.41 times for GC-including inversions) are larger than the proportion of the mutation rates (approximately 1.4 times [14]) between them. These observations suggest that the abundance of ultramicro inversions in chromosome Y is mainly subject to high diversity of the genomic structures specifically in chromosome Y [14] rather than male driven evolution. One possibility is that frequent intrachromosomal recombinations in chromosome Y [14] had been involved in frequent ultramicro inversions.

### Ultramicro inversions validated by phylogenetic profiles

**Figure 3:**
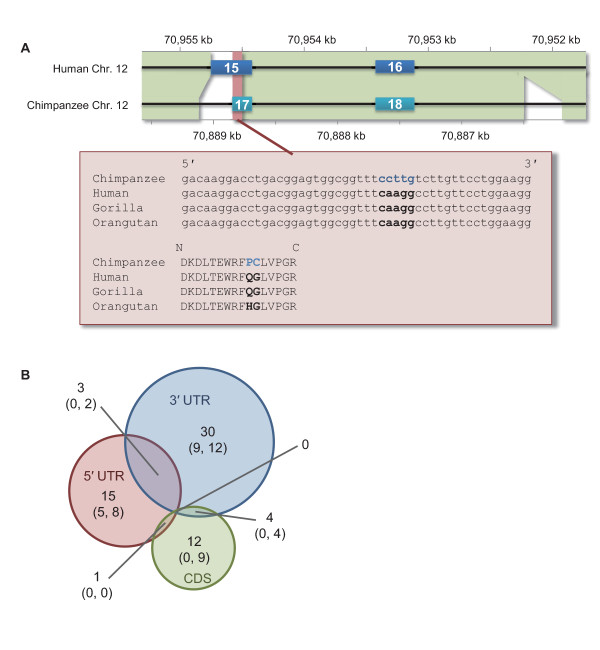
**Ultramicro inversions found within genes**. (A) The multiple alignment around the ultramicro inversions specifically identified in the chimpanzee lineage in receptor-type tyrosine-protein phosphatase beta genes (PTPRB) and the genomic structures of a part of PTPRB transcripts in the human (HIT000321866 from H-InvDB) and chimpanzee genomes (XM_509219 from Refseq). This inversion is included in one of the Fibronectin type III domains in a tandem array in PTPRB protein. Bold blue characters indicate the ultramicro inversion. Numbers within the boxes represent the exon numbers. The genomic regions with green and red backgrounds are subject to one-to-one alignment, and the red background corresponds to the multiple alignment. (B) Venn diagram of the ultramicro inversion frequencies in coding region sequences (CDS), 5' UTR, and 3' UTR. Numbers in parenthesis represent the ultramicro inversion frequencies that were inferred to have occurred specifically in the human and chimpanzee lineages, respectively. No ultramicro inversions in the genes showed the incomplete lineage sorting among human, chimpanzee, and gorilla.

By comparing the ultramicro inversions within the human-chimpanzee alignments with the orthologous sequences of the primate outgroups, the lineages in which the inversions occurred can be inferred (Figure 3A). Generating multiple alignments of ultramicro inversions concatenating their neighbors of human, chimpanzee, gorilla, and/or orangutan as outgroups, the species possessing the inverted sequences were identified. In 1,424 ultramicro inversions out of 2,377, the lineages in which the sequences had inverted were definitely determined (Table 1). Four hundred and seventy-nine and 911 inversions were identified specifically in the human and chimpanzee sequences, respectively, suggesting that they had occurred specifically in the human and chimpanzee lineages after the separation between the two species. On the other hand, 34 inversions appeared to be inconsistent with the species phylogeny among human, chimpanzee, and gorilla, suggesting incomplete lineage sorting in the common ancestor of these three species: 20 ultramicro inversions shared between human and gorilla and 14 between chimpanzee and gorilla (Table 1). The former represented the gene phylogeny as ((Human, Gorilla), Chimpanzee) and the latter represented the gene phylogeny as (Human, (Chimpanzee, Gorilla)).

While our detection method for ultramicro inversions possessed a high degree of accuracy, it is noteworthy that these 1,424 ultramicro inversions were also supported by the phylogenetic profiles of the outgroups. Thus, we considered that these inversions were very plausible. Out of the rest of 953 ultramicro inversion, we could not obtained the strong support by phylogenetic profiles in 652 ultramicro inversions and the orthologous sequences of gorilla or orangutan in 301 ultramicro inversions.

### Ultramicro inversions within genes

To examine the impact of ultramicro inversions on gene evolution in the human lineage, we searched for ultramicro inversions within those exons defined in H-InvDB [15], and found a total of 65 inversions (Figure 3). More than half the inversions were identified in the 3' UTR region. Although 17 ultramicro inversions out of 65 were found in the coding regions, they were either inferred to have occurred in the chimpanzee lineage specifically (13 inversions) or not supported by the phylogenetic profiles (four inversions) (Figure 3B). The 17 genes of which ultramicro inversions were identified in the coding regions included several well-annotated ones such as tumor protein p73 (TP73), protein tyrosine phosphatase receptor type B (PTPRB), and NADPH oxidase organizer 1 (NOXO1). In 16 out of 17 inversions, biochemically different amino acids were observed between human and chimpanzee. These inversions ranges from five to 24 bp and affected the corresponding amino acid sequences from two to nine residues. In the remaining one, a stop codon was observed in the human sequence but not in the chimpanzee sequence: only four amino acids were extended in the chimpanzee sequence. These observations suggest that ultramicro inversions in coding regions have contributed to gene evolution mainly in the chimpanzee lineage.

## Discussion

We developed a highly sensitive and distinctly specific method for identifying ultramicro inversions hidden within nucleotide alignments. This method could be very effective for sequences with average base compositions of the human and chimpanzee genomes as well as would work well for those with extremely biased base compositions such as 100% AT content (Figure 1) with extra filtering for simple repeats. Positive predictive values (number of true positives/(number of true positives + number of false positives)) are more than 0.9998 for the former case and 0.993 for the latter case, respectively. However, this method is remarkably less sensitive for the extra-short ultramicro inversions consisting of 5 or 6 bp (Figure 1). There may be two plausible reasons. One is the word size of the homology search for inverted sequences. Our method involved using the BLAST program blastn with a word size of five to detect inversions, which sometimes failed to capture the extra-short inversions. The other possibility is limitations in the detection of difference-rich regions. Our method focused on the alignment region in which a trio of difference signatures, mismatches, and gap blocks were neighbors. Some inverted segments can be forwardly aligned with fewer than three differences (e.g., inversions in palindromes), which is beyond our criteria. The frequency of hidden inversions increases as the size decreases. Nevertheless, as described above, this method is useful in identifying ultramicro inversions with a high degree of specificity.

In addition to macroscopic and microscopic inversions, a large number of ultramicro inversions, ranging from five to 125 bp, were detected between the human and chimpanzee genomes using our method (Table 1). From this observation, we defined the size of ultramicro inversions equal to or less than 125 bp. Based on the simulations, at most approximately 100 false positives (4% of the total) were expected. On an average, 0.983 ultramicro inversions were found per Mb of the human-chimpanzee alignments. These inversions had been treated as mismatches and gaps in the local alignment, suggesting that the identification of ultramicro inversions is one of the effective ways for fine-tuning the local alignments. However, we found only 0.0319% and 0.126% of mismatches and gaps in the whole human-chimpanzee genome alignments were derived from the ultramicro inversions, respectively. The nucleotide divergence between chimpanzees and humans before and after excluding the ultramicro inversions was estimated at 0.013276 and 0.013271, respectively, indicating that ultramicro inversions hardly affect the nucleotide divergence between human and chimpanzee. Still, because of the relatively low sensitivity in detecting extra-short and palindrome-like inversions, the number of ultramicro inversions may be greater within the human-chimpanzee alignments. One of our most important findings was the large fraction of AT-exclusive ultramicro inversions (Figure 2A). Our method included additional filtering of AT-exclusive inversions, which excluded inversions consisting of mono- or dinucleotide repeats of A and T. The simulation produced indicated a very high positive predictive rate. However, some of the AT-exclusive inversions may have been false positives because of the unknown aspects of genomic evolution. Filtering inversion candidates using the phylogenic profile would generate a highly specific subset of inversions [5]. The high frequency of odd-length inversions in nearly minimum size (Figure 2A) would be independent of the inversion identification algorithm. The simulation indicated that our algorithm did not perform better the ultramicro inversions in odd numbers (Figure 1). In addition, high frequency of odd-length inversions was observed in both odd and even numbers of the word size for BLASTN (data not shown), suggesting that this is independent of the homology searching algorithms. These observations implied that high frequency of odd-length inversions in the human and chimpanzee genomes would be involved in undefined biological causes such as the structure of DNA strands for generating inversions.

By comparing inverted segments with the primate outgroup, 206 of the AT-exclusive inversions belonged to this specific subset (Table 1), still suggesting frequent AT-exclusive inversions. The inversions ranging from 23 to 125 bp could be any one of the ultramicro inversions hidden in a local alignment or small-size inversions identified as a single or a string of local alignment [6]. Size distribution of the inversions roughly indicated that inversions less than 40 bp were preferably hidden in the local alignments between the human and chimpanzee genomes (Additional File 1: Figure S1). The ultramicro inversions are also distinguished from larger microscopic inversions that are detectable as a single alignment or a string of local alignments, in that the exact boundaries of ultramicro inversions can be identified easily within the local alignment. This may have a significant insight into the elucidation of the mechanism for the ultramicro inversions.

In this study, the human-chimpanzee alignments were generated by the G-compass pipeline [12]. Although the G-compass pipeline is different from the UCSC axtNet alignment [16] based on the definition of orthologous alignments, both methods initially generate local alignments with blastz [16] or its successor lastz [17]. Thus an equivalent number of ultramicro inversions is likely to be obtained from the UCSC axtNet alignment. As expected, 2,364 ultramicro inversions were found in the human-chimpanzee alignments using UCSC axtNet alignment, suggesting that most of the ultramicro inversions are independent of the G-compass pipeline. Although we have not examined for ultramicro inversions within the genome alignment generated by local alignments other than blastz, differences in the ultramicro inversions between different alignment algorithms may be helpful in verifying the behavior of the alignment algorithms involving ultramicro inversions either erroneously aligned or excluded from the local alignments.

Out of the 1,424 ultramicro inversions validated by phylogenetic profiling, 911 were found to have occurred specifically in the chimpanzee lineage, which were approximately twice more than those (479) in the human lineage (Table 1). Several studies have indicated that the sequence accuracy of the chimpanzee genome is poorer than that of the human genome [18,19] because of the lower coverage. This may be one of the causes of the abundance of ultramicro inversions in the chimpanzee lineage. However, the substitutions especially those in the chimpanzee lineage were at most 1.05 times more than those in the human lineage [19], indicating that a large number of ultramicro inversions in the chimpanzee lineage were unlikely to be the result of sequence errors. Higher level of false assembles of the sequence reads in the chimpanzee genome than the human's might be another explanation. It can be a cause for the false positives in the larger inversions as a single alignment or a string of local alignments (microscopic inversions) than ultramicro inversions. However, this may be also difficult to explain ultramicro inversions within a local alignment. Thus, the differences in inversion frequencies between humans and chimpanzees give an insight into the different histories of genomic structural changes between the two species. Furthermore, this observation ensures the abundance of ultramicro inversions in coding regions found specifically in the chimpanzee lineage. As shown in Figure 3A, ultramicro inversions substitute more than one amino acid at a time into physicochemically different ones. The inversion in PTPRB genes in chimpanzee (Figure 3A) had altered a string of two residues of glutamine and glycine into physicochemically different ones, proline (residue 1229) and cysteine (residue 1230), respectively. In contrast, the hydrophilic residue of glutamine or histidine at the corresponding site to the residue 1229 is conserved across amniotes, and the glycine at the corresponding site to the residue 1230 is conserved across tereosts and tetrapods. This implies that the ultramicro inversion had altered the function of the corresponding fibronectin type III domain. This implies that such ultramicro inversions played a role in drastic protein evolution in the chimpanzee lineage.

**Figure 4:**
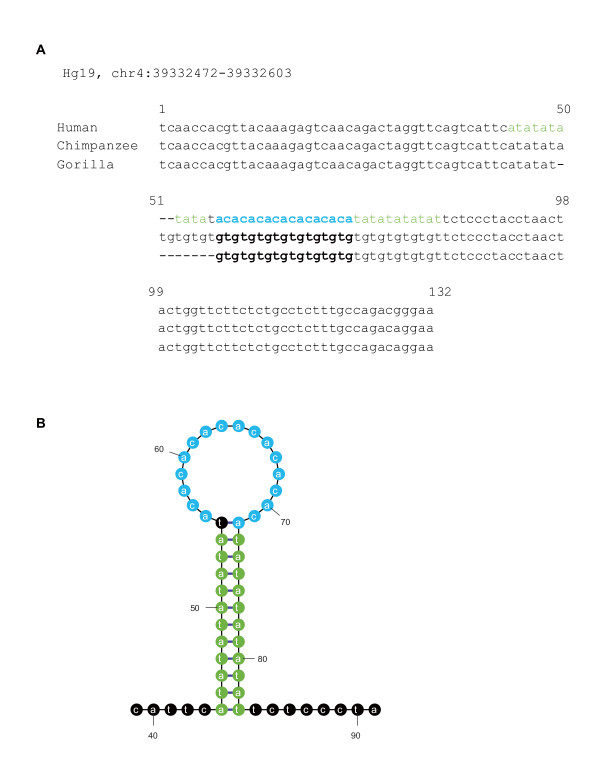
**Candidate of cruciform-mediated ultramicro inversions**. (A) Nucleotide alignments of human chromosome (chromosome 4: 39332472-39332603) and its orthologous sequences to the chimpanzee and gorilla genomes including the ultramicro inversions sandwiched by inverse repeats. Characters in bold-blue and green represent ultramicro inversions and inverted repeats, respectively. The inverted repeat at the 3' end of ultramicro inversions may have been inserted by cruciform-formation following inversion. (B) One strand of cruciform-DNA inferred by the Mfold program [25]. Characters highlighted in blue and green represent the ultramicro inversion and inverted repeats, respectively.

Although it has not been clear how ultramicro inversions have occurred, our findings of frequent ultramicro inversions in chromosome Y and the AT-exclusive regions suggests that ultramicro inversions are preferably located in those genomic regions that may relate to genomic instability. To examine the relationship between ultramicro inversions and genomic instability in detail, we compared the positions of ultramicro inversions with those of the genomic features involved in stability of the human genome. Ultramicro inversions significantly and frequently overlapped on the boundaries of *L1 *and *Alu *(*p *< 0.001) and were located in the region (<100 bp) closer to these transposable elements (*p *< 0.001), strongly suggesting that ultramicro inversions are associated with the genomic features generating instability as previously indicated by the macroscopic inversions [20]. To elucidate the mechanisms of ultramicro inversions at the molecular level, we examined the genomic features near the inverted segments and found that cruciform formation with inverted repeats mediated the ultramicro inversions, indicating that inverted repeats on both ends of the inversion segments were the signature of the cruciform-forming inversions [21]. We found 52 ultramicro inversions sandwiched between the inverted repeats of ≥10 bp, out of which 38 were the GC-including inversions. This represents a small fraction of the total ultramicro inversions but is significantly greater than expected (p < 0.001), suggesting that the ultramicro inversions were partly generated via cruciform formation. The ultramicro inversion in Figure 4 is sandwiched between inverted repeats and was possibly generated via the cruciform formation. The inverted repeat next to the 3' end of the ultramicro inversion segment is specifically found in the human sequence. This implies that inversion follows double-strand breaks, strand displacement by the invading 3' end, *de novo *DNA synthesis, and concomitant DNA elongation [21]. Thus, we have shown that ultramicro inversions are related to the genomic features involved in genomic instability, which is a characteristic similar to that of macroscopic inversions.

## Conclusions

We developed an effective method for identifying ultramicro inversions within pairwise alignments and found a large number of ultramicro inversions within the local alignments between the human and chimpanzee genomes. This is the first finding of an abundance of short and extra-short inversions within the local alignments between closely related species. This observation suggests that a considerable number of ultramicro inversions could be found within the alignments between individuals from different populations. Furthermore, some of the adjacent SNPs may be ultramicro inversions as well as large inversions observed in HapMap data [4]. Identification of ultramicro inversions within human populations may be helpful in elucidating how phenotypic characteristics have diversified during human evolution. While our inversion-identification method was helpful for examining the impact of microscopic inversions, this method is also applicable in fine tuning genome alignments by distinguishing ultramicro inversions from nucleotide substitutions and indels.

## Methods

### Identification method for ultramicro inversions

Ultramicro inversions were detected within a local pairwise alignment by the following two procedures: identifying difference-rich regions and searching for inverted regions in these difference-rich regions. A region rich in mismatches and gaps was initially detected as a trio of the nearest mismatches and gap blocks which were more closely positioned on an alignment than expected. Each trio consisted of either three mismatches, two mismatches and one gap block, or one mismatch and two gap blocks that were located in different sequences of a pair. The trio was extracted by scanning the pairwise alignment. When a mismatch or gap block was found and the next two mismatches and/or gaps were located within a region of *n *- 1 consecutive sites, the conditional probability of the trio within *n *sites *P*_trio_(*n*) (*n *≥ 3) was calculated by the equation given below,

$$P_{\text{trio}}\left(n\right)=1-{\left(1-p_{{}_{\text{d}}}\right)}^{n-1}-\left(n-1\right)p_{{}_{\text{d}}}{\left(1-p_{{}_{\text{d}}}\right)}^{n-2}$$

where *p*_d _represents the average number of mismatches and gap blocks per site.

During the detection process, some parts of the inversions were found to be aligned without mismatches, as follows:

The inversion of 8 bp included a palindrome in part and was aligned with the palindrome. We called this a partially palindromic inversion. In order to identify this kind of inversion, we searched for the region where an identically aligned region was sandwiched by two gap blocks inserted in different sequences of pair. The conditional probability of the two gap blocks within *n *sites *P*_duo_(*n*) (*n *≥ 2) is given as follows:

$$P_{\text{duo}}\left(n\right)=1-{\left(1-p_{\text{g}}\right)}^{n-1}$$

where *p*_g _is the average number of the gap blocks per site.

We extracted such trios and duos where *P*_trio_(*n*) < 0.05 or *P*_duo_(*n*) < 0.05. These trios and duos were merged and then extended with 50 bp at both ends. The resultant regions were subject to subsequent analysis.

The inverted regions were detected within the difference-rich regions described above using the NCBI BLAST program blastn [22]. For blastn, the word size was set at five, and the query sequences were not filtered (-p blastn -F F -W 5). If an inverted alignment region largely or completely overlapping the corresponding forward alignment, and if the inversion was aligned with higher similarity than the corresponding forward alignment based on following the criteria stated below, we defined the inverted region as an ultramicro inversion. (i) For the region based on *P*_trio_(*n*) < 0.05, the inverted alignment region was completely included within the forward alignment region, or >80% of the inverted region overlapped with the corresponding forward alignment region whereas the rest of the inverted region was aligned as gaps in the forward alignment. Similarity of the inverted alignment was >0.95, which was >1.25 times higher than that of the forward alignment. The corresponding forward alignment included the trio. (ii) For the region based on *P*_duo_(*n*) < 0.05, the corresponding region of the forward alignment consisted of a duo of gap blocks described above, and a region sandwiched by the gaps was identically aligned with >50% of the segment. Similarity of the inverted alignment and coverage of the inverted region to the forwardly aligned region were 100%. Following these procedures, we excluded the some part of AT-exclusive inversions which could be explained by the other mechanisms than inversions. One of the inversions to be excluded consisted of mononucleotide repeats of A and T such as 5'-AAATTTTTTT-3': the inversion 5'-AAAAAAATTT-3' could be explained by with stretch and shrink of the repeats. The other consisted of staggered AT dinucleotide repeats such as 5'-ATATATATATA-3': the inversion 5'-TATATATATAT-3' could be explained by insertion of deletion of A or T. The length of ultramicro inversions was defined as the length of the inverted segments determined by the blastn program.

### Simulation

In order to evaluate the power of our identification method, simulations were performed using sequence alignment sets of random sequences, each consisting of 100,000 pairs of around 5,000 bp sequences and including an inversion in each pair. Each sequence pair was generated by the sequence evolution simulator Indelible [13], allowing insertions and deletions (indels) from a random sequence of 5,000 bp, assuming the HKY sequence substitution model [23], the indel lengths distributed with the Lavalette distribution setting the decimal at 2 and the maximum indel length at 50, and the prior sequence conditions similar to the human-chimpanzee genome alignment: setting the base compositions of *g*_A_, *g*_T_, *g*_C_, and *g*_G _at 0.289, 0.304, 0.203, and 0.204, respectively, the transition/transversion ratio at 1.75, the average of sequence substitutions per site at 1.00, the indel/substitution ratio at 0.159, and the shape parameter for the gamma distribution and the number of categories for the discrete gamma approximation set at 0.65 and 5, respectively. A short region of 5 to 50 bp length in one sequence of each pair was inverted, and the pair was aligned with MAFFT [24]. The inversion lengths were fixed in all the 100,000 pairs of a sequence alignment set. We generated eleven sequence alignment sets with 5, 6, 7, 8, 9, 10, 15, 20, 25, 30, and 50 bp inversions. The other sequence alignment groups were generated for assessing the intensity of AT-exclusive sequences. The sequence models and prior sequence parameters were equal to the simulation above except for the base compositions; (*g*_A_, *g*_T_, *g*_C_, *g*_G_) = (0.5, 0.5, 0, 0). For the AT-exclusive alignments, seven alignment sets were generated, and 5, 10, 15, 20, 25, 30, and 50 bp inversions were included in the individual alignment sets. In these simulations, *p*_d _and *p*_g _were set at 0.0100 and 0.00150, respectively.

### Identification of the ultramicro inversions within the human-chimpanzee alignments

To identify ultramicro inversions within the alignments between the human and chimpanzee genomes, one-to-one (i.e., orthologous) alignments were generated between the human and chimpanzee using the hg19 human genomic sequence and panTro2 chimpanzee genomic sequence from the UCSC genome browser (http://genome.ucsc.edu/). The alignments were constructed with the G-compass pipeline [11,12] based on the blastz local alignments [16] and its unique and non-redundant reciprocal best hits. Applying the method above after setting *p*_d _and *p*_g _at 0.0136 and 0.00150 respectively, we obtained ultramicro inversions within the human-chimpanzee alignments.

The human-gorilla and human-orangutan one-to-one alignments were generated by the same procedures, using the gorGor3 gorilla and ponAbe2 orangutan genomic sequences from the UCSC genome browser. The human-chimpanzee alignments including the ultramicro inversions were grouped with the human-gorilla and human-orangutan alignments in which the human sequence overlapped the inversion segments in the human-chimpanzee alignments by a single linkage. In each group, the human, chimpanzee, and gorilla and/or orangutan sequences were multiply aligned by MAFFT [24].

The validation of ultramicro inversions was performed using these multiple alignments. In the alignment sites of the inversions, if fewer than two mismatches or gaps were found between the human and outgroup sequences and three or more mismatches or gaps were found between the chimpanzee and outgroup sequences, we concluded that the chimpanzee sequence had been inverted. The human inverted sequences were also detected in the same way. If the phylogenetic profile of the inversion was inconsistent with the species phylogeny among human, chimpanzee, and gorilla, the inversion was verified with the incomplete lineage sorting.

In order to relate ultramicro inversions to the genomic features, we used two kinds of genomic tracks available from the public database. Mapping information of exons and coding regions of human transcripts on the human genome were obtained from H-InvDB version 7.5 (http://hinv.jp/hinv/ahg-db/) [15]. Mapping information of *Alu *and *L1 *was obtained from the chromOut repeat-masking annotation files on the human genome from the UCSC genome browser (http://genome.ucsc.edu/). To determine if ultramicro inversions preferentially occur in the neighborhood of transposable elements, we conducted a 1,000 times trial of the random distribution of the short segments on the human genome. Given that the size distribution of 2,377 short segments was identical to that of the ultramicro inversions within the local alignments between the human and chimpanzee genomes, these segments were randomly distributed on the human genome. Frequency distributions of every 100 bp of genomic distances between the segment and nearest transportable element were computed. If a boundary of the mobile element was included in the ultramicro inversion, the distance was set to zero. Frequencies of the short segments in every 100 bp were counted from the 1,000 times trial of the random distribution. The value of *p *< 0.001 indicates no appearance of the short segment in the trial. We applied this procedure to determine if the inverted repeats were usually located on both ends of the ultramicro inversion. We also investigated the possibility that the inverted repeats were randomly distributed on both ends of the short segments instead of calculating the distance from the short segments to the transposable elements.

The program searchUMI.pl used for ultramicro inversion identification and written in Perl as well as the pairwise alignment data between the human and chimpanzee genomes are available from http://hinv.jp/g-compass/2011hara/index.html.

## Abbreviations

AT content: adenine and thymine content; Gb: giga base; Mb mega base.

## Competing interests

The authors declare that they have no competing interests.

## Authors' contributions

YH and TI conceived and designed the experiments, and YH analyzed the data. YH and TI wrote the paper and read and approved the final manuscript.

## Supplementary Material

Additional File 1 **Figure S1. Size distributions of ultramicro and small-size inversions**. Distributions of ultramicro and small-size inversions over different ranges of sizes in nucleotides. The blue, red, and green bars represent the numbers of GC-including and AT-exclusive ultramicro inversions and small-size inversions obtained from Feuk et al. [6], respectively. Click here for file
