# Supplementary material for: Abundance of ultramicro inversions within local alignments between human and chimpanzee genomes
Source: BMC Evol Biol. 2011 Oct 19;11:308. doi: 10.1186/1471-2148-11-308 (PMC3227671; doi:10.1186/1471-2148-11-308)
Supplement: Additional File 1 — Figure S1. Size distributions of ultramicro and small-size inversions. Distributions of ultramicro and small-size inversions over different ranges of sizes in nucleotides. The blue, red, and green bars represent the numbers of GC-including and AT-exclusive ultramicro inversions and small-size inversions obtained from Feuk et al. [6], respectively. [file 1471-2148-11-308-S1.DOC]

**Additional file 1: Figure S1.**

**Figure S1. Size distributions of ultramicro and small-size inversions.** Distributions of ultramicro and small-size inversions over different ranges of sizes in nucleotides. The blue, red, and green bars represent the numbers of GC-including and AT-exclusive ultramicro inversions and small-size inversions obtained from Feuk et al. [1], respectively.

**References**

1. Feuk L, MacDonald JR, Tang T, Carson AR, Li M, Rao G, Khaja R, Scherer SW: **Discovery of human inversion polymorphisms by comparative analysis of human and chimpanzee DNA sequence assemblies.** *PLoS Genet* 2005, **1:**e56.

**Figure S1.**
